# Retraction Note: Functional cargos of exosomes derived from Flk-1^+^ vascular progenitors enable neurulation and ameliorate embryonic anomalies in diabetic pregnancy

**DOI:** 10.1038/s42003-025-08512-y

**Published:** 2025-08-06

**Authors:** Songying Cao, Yanqing Wu, E. Albert Reece, Cheng Xu, Wei-Bin Shen, Sunjay Kaushal, Peixin Yang

**Affiliations:** 1 Department of Obstetrics, Gynecology & Reproductive Sciences, University of Maryland School of Medicine, Baltimore, MD USA; 2 Department of Biochemistry & Molecular Biology, University of Maryland School of Medicine, Baltimore, MD USA; 3 Department of Surgery, University of Maryland School of Medicine, Baltimore, MD USA; 4 Present Address: Institute of Life Sciences, Wenzhou University, Zhejiang Province 325035 Wenzhou, China; 5 Present Address: Division of Cardiovascular-Thoracic Surgery, Ann & Robert H. Lurie Children’s Hospital of Chicago, 225 E. Chicago Avenue, Chicago, IL 60611 USA

Retraction to: *Communications Biology* 10.1038/s42003-022-03614-3, published online 01 July 2022

The Editors have retracted this article at the recommendation of the University of Maryland, Baltimore (UMB). A peer-review investigation committee, overseen by the University’s Research Integrity Office (RIO) found that data reported in this article, specifically that presented in Figure 5d, was incorrectly labelled and obtained from experiments different from those described in the article. The UMB also informed the journal that the investigation committee could not confirm the integrity of the data because not all of the relevant original data was available for inspection during the investigation. The Editors no longer have confidence in the results and conclusions of this article.

The authors have not explicitly stated whether they agree or disagree with the retraction.

